# Ixodes dammini: a junior synonym for Ixodes scapularis.

**DOI:** 10.3201/eid0401.980125

**Published:** 1998

**Authors:** M Sanders


throughout the city, although many patients came from the same area.

A seroepidemiologic study to determine possible new sources of infection (e.g., dogs, cats) and estimate rates of seropositivity in cattle and sheep and a case-control study on new cases are being conducted.

**F. Pfaff,* A. François,* D. Hommel,† I. Jeanne,* J. Margery,‡ G. Guillot,† Y. Couratte-Arnaude,‡ A. Hulin,† and A. Talarmin***

*Pasteur Institute of French Guiana, Cayenne, French Guiana; †Cayenne Hospital, Cayenne, French Guiana; and ‡Army Health Care Service, Cayenne, French Guiana

### Reference

1. Floch H. La pathologie vétérinaire en Guyane française (les affections des porcins, des caprins et des ovins). Revue Elev Méd Vét Pays Trop 1955;8:11-3.

### *Ixodes dammini:* A Junior Synonym for *Ixodes scapularis*

**To the Editor:** The authors of "A new tick-borne encephalitis-like virus infecting New England deer ticks, *Ixodes dammini*" (1) provide useful information regarding a possibly new tick-borne encephalitis-like virus. However, the use of the name *Ixodes dammini* is not accurate for describing this species. *I. dammini* (Spielman, Clifford, Piesman, and Corwin) was synonymized with *Ixodes scapularis* (Say) in 1993 by Oliver et al. (2) and was redescribed in 1996 (3) to reduce confusion regarding identification. Keirans and colleagues summarize a wide array of rigorous studies involving hybridization, assortative mating, isozymes, and morphometrics, all of which provide evidence supporting the synonymization of the two tick species (3).

The synonymization of *I. dammini* with *I. scapularis* has been widely accepted. "*I. scapularis* (= *I. dammini*)" is still often used, but the use of *I. scapularis* as the sole nomen for this species is becoming more common (4). Oliver et al. (2) have established *I. dammini* as a junior subjective synonym of *I. scapularis.* If scientifically rigorous evidence exists justifying the reestablishment of the species name *I. dammini,* it must be published according to proper procedure. The proper nomenclature of any species, let alone one of such widespread notoriety and public health importance, is too important to be relegated to a footnote. Until such evidence is presented, the continued misuse of *I. dammini* serves only to confuse health-care providers, public health professionals, and lay persons.

On a secondary matter, on page 167 of the dispatch, the authors state that "*I.* (Pholeoixodes) *cookei* is a one-host tick that is only distantly related to *I. dammini* and only rarely feeds on humans or mice" (1). *I. cookei* is a three-host tick (D.E. Sonenshine, pers. comm.), as are all the members of the genus Ixodes.

**Martin Sanders**

Maryland Department of Health and Mental Hygiene, Baltimore, Maryland, USA

### References

1. Telford SR III, Armstrong PM, Katavolos P, Foppa I, Garcia ASO, Wilson ML, Spielman A. A new tick-borne encephalitis-like virus infecting New England deer ticks, *Ixodes dammini*. Emerg Infect Dis 1997;3:165-70.
2. Oliver JH, Owsley MR, Hutcheson AM, James C, Chen W, Irby S, et al. Conspecificity of the ticks *Ixodes scapularis* and *Ixodes dammini* (Acari: Ixodidae). J Med Entomol 1993;30:54-63.
3. Keirans JE, Hutcheson HJ, Durden LA, Klompen JSH. *Ixodes scapularis* (Acari: Ixodidae): redescription of all active stages, distribution, hosts, geographical variation, and medical and veterinary importance. J Med Entomol 1996;33:297-318.
4. Centers for Disease Control and Prevention. Lyme disease—United States, 1996. MMWR Morb Mortal Wkly Rep 1997;46:531-5.

### The Name *Ixodes dammini* Epidemiologically Justified

**To the Editor:** Although a large body of evidence has been interpreted as supporting conspecificity of the deer tick (*Ixodes dammini*) and the blacklegged tick (*Ixodes scapularis*), according to Chapter VI, Article 23 L of the International Code of Zoological Nomenclature (1), "A name that has been treated as a junior synonym may be used as the valid name of a taxon by an author who considers the synonymy to be erroneous...."

Current use of *I. scapularis* to refer to the vector of Lyme disease obscures important epidemiologic issues. One of the reasons for "sinking" *I. dammini* was to make it easier to diagnose Lyme disease in areas where the disease was thought to be nonendemic: "The belief that *I. dammini* does not occur south of Maryland and that *I. scapularis* is a separate and

